# The geospatial and conceptual configuration of the natural environment impacts the association with health outcomes and behavior in children and adolescents

**DOI:** 10.1186/s12942-022-00309-0

**Published:** 2022-08-11

**Authors:** Carina Nigg, Claudia Niessner, Alexander Burchartz, Alexander Woll, Jasper Schipperijn

**Affiliations:** 1 Institute of Sport Science, University of Bern, Bremgartenstrasse 145, 3012 Bern, Switzerland; 2 Institute of Sports and Sports Science, Karlsruhe Institute of Technology, Engler-Bunte-Ring 15, 76131 Karlsruhe, Germany; 3 Department of Sports Science and Clinical Biomechanics, University of Southern Denmark, Campusvej 55, 5230 Odense, Denmark

**Keywords:** GIS, Green space, Blue space, Physical activity, Mental health, Physical fitness, Cohort study, Youth

## Abstract

**Background:**

Studies investigating associations between natural environments and health outcomes or health behaviors in children and adolescents yielded heterogenous results to date. This may be the result of different geospatial configurations of the natural environment and confounding characteristics of the study population. Thus, we investigated how the relationship between the natural environment and mental health, muscular fitness, and physical activity varies depending on the geospatial configuration of nature and children’s and adolescents’ characteristics.

**Methods:**

Data were derived from the German Motorik-Modul (MoMo) cohort study (2018–2020) that investigates physical activity, muscular fitness, and health parameters in a national sample of children and adolescents (*N* = 2843) between four and 17 years (*M*_age_ = 10.46 ± 3.49 years; 48.3% girls). Mental health was assessed via questionnaire, muscular fitness via standing long jump, and physical activity with 7-day accelerometer measurement. Using geographic information systems, land cover, and land use data, three different nature definitions were applied. Both circular buffers (100–1000 m) and street-network buffers (1000–5000 m) were created for each of the nature definitions. Associations were explored with linear regression models, and interaction analysis was used to investigate how those relationships vary by gender, age, and socio-economic status.

**Results:**

The relationship between the three outcomes and the natural environment varied considerably depending on the nature definition, buffer size, and buffer type, as well as socio-demographic characteristics. Specifically, when comparing youth with a high socio-economic status to those with a medium socio-economic status, smaller circular buffer distances were related to less physical activity, but larger street-network buffer distances were related to greater mental health problems. Distinct relationships also occurred for youth with low socio-economic status in those relationships, with the pattern being less clear.

**Conclusions:**

For future health research studies that investigate the role of the natural environment, we argue for the development of an a-priori model that integrates both geospatial considerations (nature definition, buffer type, and buffer size) and conceptual considerations (health outcome/behavior, sample characteristics) based on potentially underlying mechanisms that link the natural environment and the health outcome or behavior under investigation to theoretically underpin the geospatial configuration of the natural environment.

**Supplementary Information:**

The online version contains supplementary material available at 10.1186/s12942-022-00309-0.

## Background

Childhood and adolescence are sensitive developmental periods, which makes it important to identify determinants that prevent mental illness [1] and foster physical activity and physical health [2], in this way promoting that children and adolescents flourish and become healthy adults. In the light of rapidly changing environments due to urbanization and climate change, the environment, especially green space, have been increasingly recognized as an important factor and determinant of health and health behavior [3], specifically for physical activity [4, 5], physical health [6], and mental health [7]. Theoretically, green space in the form of parks and trails, constitutes attractive opportunities to engage in physical activity, such as active play or bicycling [8]. Multiple conceptual models exist that connect exposure to green space and mental health, including mechanisms via ecosystem services (e.g., reduced air pollution and heat), psychological benefits (e.g., reduced stress and affective restoration), and health behaviors (e.g., social interactions and physical activity) [9–11] as well as physical health, e.g. via pathways of reduced air pollution, noise, and temperature [6].

However, while findings regarding green space and physical health in children and adolescents are limited to date [12], findings regarding associations between green space, mental health, and physical activity in children and adolescents are highly heterogenous: a recent systematic review found inconsistent associations between green space (distance to, count/proportion, or type of green space) and different physical activity domains and well-being [13]. These heterogenous findings may be explained by prevailing methodological issues.

Methodologically, there has been no consensus on how to assess the built environment via geographic information systems (GIS) in health research. For example, a comprehensive review of GIS derived built environment measures in physical activity research showed large variability and a lack of definition of built environment variables [14], hindering comparisons across studies. Also, when looking specifically at studies investigating GIS-derived green space in relation to physical activity and mental health in children and adolescents, assessment methods were highly heterogeneous regarding buffer sizes, ranging from 50 to 8050 m, buffer type, including network distance to green space as well as circular buffers for the proportion of green space within a certain area, and green space type [15].

Additionally, in the geographical literature, the problem of the relationship between a spatial variable and the outcome of interest being dependent on the spatial unit has been recognized as modifiable areal unit problem (MAUP) [16, 17]. The MAUP consists of both the scale problem, referring to different and arbitrary sizes of spatial aggregation (e.g., aggregating green space data within a 500 m vs. a 1000 m buffer), and the zone problem, referring to the configuration of the spatial zone (e.g., application of administrative boundaries vs. individual-level buffers) [16]. The MAUP has also been observed when examining geographical contexts in health research: in a study with adults, the relationship between built environment variables (e.g., mixed land use, pedestrian infrastructure) and active transport varied by buffer size and type, with this variation being inconsistent across the built environment variables, thus making it challenging to select an ideal geographical scale that fits all [18]. Similar results were obtained when examining associations between different accelerometer measures of adult’s physical activity and selected built environment measures [19], children’s active school travel behavior [20], and when investigating green space in relation to outdoor physical activity [21]. Considering mental health, the relationship between neighborhood socio-economic deprivation and the purchase of psychiatric medication was dependent on the geographical assessment of the neighborhood via micro-area, parishes, or postal codes [22]. Regarding physical health, parameters of walkability showed heterogenous relationships with obesity depending on the geographical scale being used, and the best model fit was achieved when different geographical scales for each parameter were included [23].

However, although some studies investigated how the choice of the geographical scale influences the association with a specific health parameter or health behavior, there is a lack of studies that (i) investigated different operationalizations of green space and used various buffer sizes and buffer types in children and adolescents (ii) explored variations across different health domains, and (iii), took sociodemographic characteristics of the sample for those variations into account. Hence, this study aims to investigate

1. How the relationship between green space and physical activity, mental health problems, and physical health varies by nature operationalization, buffer type, and buffer size, and
2. How these variations differ across age, gender, and socio-economic status.

## Methods

Data was obtained from the Motorik-Modul Study (MoMo). The MoMo study applies a cohort-sequence design to investigate physical fitness, physical activity, and health indicators in children and adolescents between four and 17 years in Germany [24]. For this study, we only used cross-sectional data from the latest Wave 3.1 (2018–2020) as this was the only study wave for which address data of the participants could be obtained.

### Participants and procedures

The participants for the MoMo study Wave 3 (2018–2022) were selected based on a nationwide multi-stage sampling approach with two evaluation levels to maximize representativeness [25]: first, a systematic sample of 167 primary sampling units was selected from an inventory of German communities stratified according to the classification system that measures the level of urbanization and geographic distribution [26]. The probability of any community being picked was proportional to the number of citizens younger than 18 years in that community. Second, based on the official registers of local residents, an age-stratified sample of randomly selected children and adolescents was drawn.

Due to the Covid-19 pandemic, data could not be collected at all 167 sampling points but had to be interrupted after 128 sampling points were completed. All data used in this study (Wave 3.1; 2018–2020) had been collected prior to the first Covid-19 related lockdown in March 2020. Participants were invited to examination rooms within proximity to their homes for measurement purposes. Study participation was voluntary, and participants’ guardians provided written consent. For children under the age of 11 years, parents were asked to fill in the questionnaire together with the child. The study was conducted in accordance with the Declaration of Helsinki. Ethics approval was obtained by the ethics committee of the Karlsruhe Institute of Technology. The Federal Commissioner for Data Protection and Freedom of Information was informed about the study and approved it.

### Measures

#### Sociodemographic characteristics and body-mass-index (BMI)

Participants reported age, gender, and socio-economic status. The socio-economic status is a multidimensional score based on information of both parents regarding occupational status, education, and net income, which is computed based on the procedures of Lampert et al. [27]. Based on the score quintiles, a three-level variable was created (socio-economic status low: first quintile; medium: second to fourth quintile; high: fifth quintile). Height and weight were assessed by trained staff and BMI categories were established based on the cut-off points of the International Obesity Task Force [28, 29].

#### Nature types and buffer development

First, all address data of MoMo Wave 3 were geocoded using the address batch of the Federal Agency for Cartography and Geodesy [30]. Second, the digital land cover dataset DE-LBM2018 in vector format was obtained from the Federal Agency for Cartography and Geodesy, containing information about land cover and land use. Land cover information is based on multitemporal image data (mainly RapidEye: 5 m ground resolution, 5 channels). Land use information was obtained from the ATKIS Basis-DLM about settlements, traffic, vegetation, and water bodies with a minimum mapping area of one hectare. Both land use and land cover data were transformed to comply with the European CORINE Land Cover classification (CLC) by the Federal Agency [31]. Based on this data, we developed three different indices: (1) nature index, which contains both green space and blue space, (2) green space index, which includes only vegetated areas and excludes water bodies and non-vegetated areas; and (3) accessible green space, which excluded agricultural areas. The appropriate CLC-classification for each index was selected and transformed into a raster dataset, using the cell assignment type maximum combined area and a cell size of 10 m × 10 m.

We decided for the definition of these three indices based on conceptual considerations. One index should represent the natural environment as a whole, thus including all outdoor areas that allow individuals to be exposed to any elements of nature [32], hence including both green space (e.g., urban green space, agricultural areas, forests) as well as semi-natural areas (e.g., beaches, rocks) and blue space (e.g., wetlands and water bodies) in the geospatial configuration. Although health research on blue space is still in its infancy, previous study results indicate that green space and blue space may have different relationships with health outcomes [33–36]. Thus, one nature index was concentrated on green space as this is a frequently used indicator in health research [37, 38]. The third nature indicator, accessible green space, was created with a special focus on the usability of green space, as this may be especially relevant for physical activity and muscular fitness. Hence, agricultural areas were excluded as they are often not accessible [39].

All nature indices calculations were conducted with ArcGIS Pro (version 2.6.3). Next, circular buffers with Euclidean distances from 100 m, 250 m, 500 m, and 1000 m were created around each participant’s home address. To compute street-network buffers, we obtained additional geographical data from the Federal Agency for Cartography and Geodesy (Basis-DLM) that contains topographical objects with an accuracy of ± 3 m for streets and paths [40]. For our purposes, we created a dataset that excluded motor highways and federal streets as they are only accessible with a motorized vehicle and thus could not be used in a physical activity context. Next, we computed street-network buffers using the Service Area Solver within the Network Analyst extension of ArcGIS Pro for the distances 1000 m, 3000 m, and 5000 m. The “high precision” polygon generation option was applied with a trim distance of 50 m and allowing overlap.

**Fig. 1:**
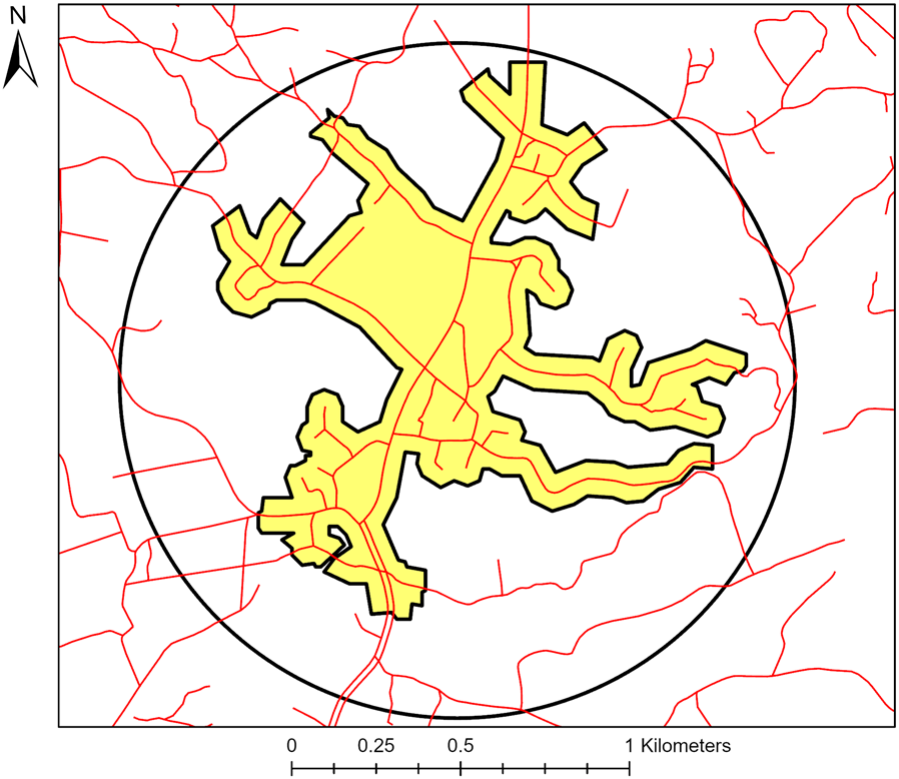
HYPERLINK "sps:id::fig1||locator::gr1||MediaObject::0" Example of a circular buffer and street-network buffer with a buffer distance of 1000 m, respectively. The area within the black circle shows the area that is considered for the natural environment if a 1000 m circular buffer distance is used, the yellow-colored area shows which area is considered for the natural environment if a 1000 m street-network buffer is used. The red lines represent streets and paths. Geobasisdaten: © GeoBasis-DE/BKG (2020). User conditions: https://sg.geodatenzentrum.de/web_public/nutzungsbedingungen.pdf

Both the circular and street-network buffers were intersected with each of the land cover included in each index to obtain the percentage of natural land cover within the specified buffer distance (see Fig. 1).

#### Moderate-to-vigorous physical activity

We decided for moderate-to-vigorous physical activity (MVPA) as a crucial health behavior during childhood and adolescence due to its numerous health benefits [41, 42] and as the built environment has been shown to relate to children’s and adolescents’ physical activity [13].

Details about accelerometry use in the MoMo study are elsewhere available [43]. Briefly, participants between 6 and 17 years were asked to wear an ActiGraph GT3x+ or ActiGraph wGT3X-BT accelerometer for 7 consecutive days. As not all participants agreed to wear an accelerometer, those associations could only be explored in a sub-sample. Participants were instructed to place the accelerometer on the right hip and to wear it during waking hours. Data was sampled with a frequency of 30 Hz. Downloaded data was converted into one-second-epochs and re-integrated into 15-s-epochs. Non-wear times were detected based on the Choi-algorithm [44]. To be considered a valid accelerometer dataset, participants had to wear the device for more than eight hours on at least 4 weekdays and 1 weekend day. To determine MVPA, two cut-off point systems were applied that are commonly used for the specific age groups, i.e., Evenson cut-offs for 6-to-10 year-olds [45] and Romanzini cut-offs for 11-to-17 year-olds [46].

#### Muscular fitness

We used the single item standing long jump to assess muscular fitness as this has been suggested to be a good general index of muscular fitness in youth [47] and this has been frequently applied in this age group [48]. Participants were standing behind a starting line with their feet together. They were asked to push forward vigorously, jump as far as possible, and land with both feet. The distance was measured from the starting line to the back of the heel closest to the starting line. Each participant jumped twice, with the maximum score (centimeters) being retained [49].

#### Mental health problems

Mental health problems were assessed using the German version of the Strength and Difficulties Questionnaire [SDQ; 50], which is an established assessment tool for mental health problems in children and adolescents [51]. The SDQ consists of five subscales: emotional symptoms, conduct problems, hyperactivity/inattention, peer relationship problems, and prosocial behavior. All subscales consist of five items with response options ranging from 0 (“Does not apply”) to 2 (“Does apply”). For this study, we only worked with the overall SDQ scale, consisting of all scales but the prosocial behavior scale. A sum-score was created, with higher scores indicating greater mental health problems. SDQ total difficulty scores were constantly related to an increased odds of clinical mental disorders in a population sample of children and adolescents [52] and are sensitive to identify individuals with clinically significant mental disorders [53]. Validity and reliability have been reported [50, 54].

#### Statistical analysis

All statistical analyses were conducted in R (version 4.1.2) [55]. To explore the association between the different indices, buffer types, and buffer sizes, we used multiple linear regression. First, we examined the distribution of the outcome variables. Visual examination confirmed no substantial distribution from normality. Second, for each nature index type as well as buffer size and type, we ran one multiple linear regression model for each outcome (MVPA, standing long jump distance, and SDQ score), respectively. Our main interest was the association between nature buffer type and size and the outcome. Based on previous findings, we considered gender, socio-economic status, age, and BMI as covariates in each model [56–59] (see also Additional file 1: Text S1). Third, as previous research has shown inequalities in the use of and access to natural environments [60, 61] as well as in the association between built environment variables and health outcomes [62, 63], we calculated interactions between the natural environment predictors and socio-demographic indicators (age, gender, and socio-economic status). In addition, for the outcome MVPA, we distinguished between weekdays (Monday–Friday) and weekend days (Saturday–Sunday) as physical activity patterns may differ due to structural changes [64]. For all models, we investigated model parameters and potential model assumption violations using the package “see” (version 0.7.0) [65].

## Results

### Descriptive results

Overall, 2843 children and adolescents between 4 and 17 years participated in the MoMo study Wave 3.1 between 2018 and 2020, which was the sample that was considered to analyze associations between natural environments and standing long jump distance as well as natural environments and mental health problems. Participants were on average 10.46 (SD = 3.49) years old, 48.3% were female, 15.1% were categorized as overweight or obese, and 19.5% were categorized as youth with low socio-economic status. We included only those participants in the analysis that had complete data on all variables including co-variates, resulting in N = 2493 for standing long jump distance and N = 2341 for mental health problems.

For the accelerometer sub-sample, 949 children and adolescents between 6 and 17 years provided valid accelerometer data. Participants were on average 11.22 (SD = 3.34) years old, 49.8% were female, 14.5% were categorized as overweight or obese, and 17.8% as participants with low socio-economic status. We included only those participants in the analysis that had complete data on all variables including co-variates, resulting in N = 923.

A detailed overview of socio-demographic information, weight status, and study variable descriptive results can be found in the supplementary material for each of the specific samples that were included in the final analysis (Additional file 1: Table S1).

### Natural environment and moderate-to-vigorous physical activity (MVPA)

As our main interest was to explore associations between different nature indices, buffer types, and buffer sizes and the respective health outcomes or behavior, we only report the unstandardized regression coefficient for the fully adjusted models. More detailed information can be obtained in Additional file 1: Tables S2–S5).

**Fig. 2:**
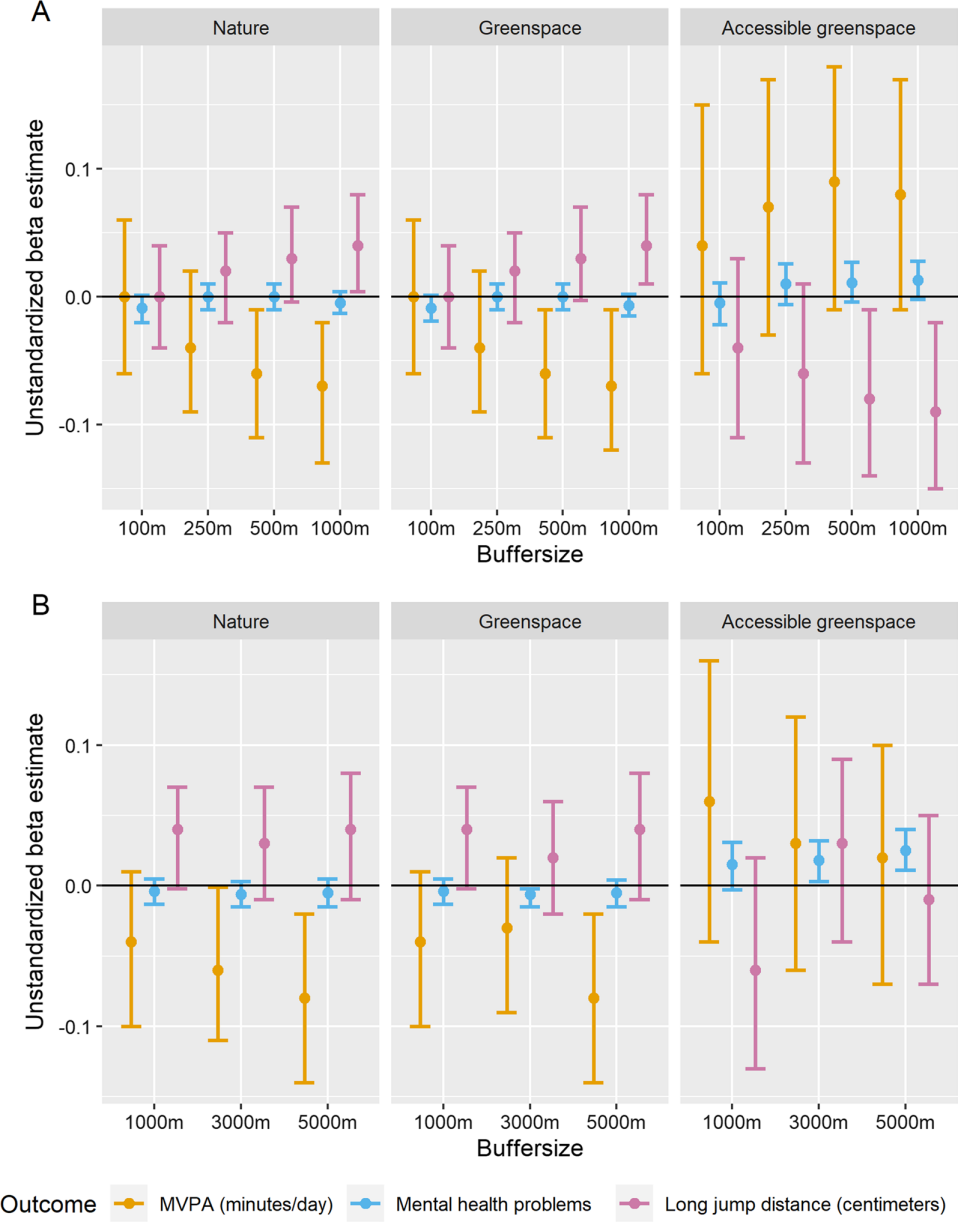
Variations of unstandardized beta regression coefficients of each nature index, buffer type, and buffer size across health outcomes. **A** Unstandardized beta estimates for circular buffers. **B** Unstandardized beta estimates for street-network buffers. Sample size: N = 923 for MVPA; N = 2493 for standing long jump distance; N = 2341 for mental health problems. Error bars represent 95% confidence intervals. All models were adjusted for age, gender, BMI, and socio-economic status. Please see Additional file 1: Table S6 for estimates of the co-variates

Multiple regression analysis revealed a heterogenous picture regarding the association between the natural environment and physical activity. More specifically, the nature and green space indices were negatively associated with MVPA for the 500 m and 1000 m circular buffer as well as for the 3000 m and 5000 m street-network buffer, indicating that more natural environment relates to less MVPA. However, none of the accessible green space buffer types or distances were associated with MVPA (see Fig. 2).

Next, we calculated interactions to explore potential variations based on individuals’ characteristics. The results showed that the relationship varied by socio-economic status: compared to youth with medium socio-economic status, nature within circular buffer distances from 100 to 250 m was consistently related to lower MVPA in youth with higher socio-economic status across the three nature definitions. This was not observed for youth with low socio-economic status. However, the 3000 m and 5000 m accessible green space street-network buffer distances were negatively related to youth’s MVPA with lower socio-economic status compared to youth with medium socio-economic status. This was not observed for youth with high socio-economic status (see Additional file 1: Fig. S1).

When distinguishing between weekday and weekend MVPA, negative associations were observed between nature circular (250–1000 m) and street-network buffers (1000–5000 m), green space circular (500–1000 m) as well as green space street-network buffers (1000–5000 m) and weekday MVPA, but not with weekend MVPA. None of the accessible green space buffer types and sizes was related to MVPA neither on weekdays nor on the weekend (see also Additional file 1: Fig. S2).

### Natural environment and muscular fitness

Distinct relationships also occurred for standing long jump: while both the nature and green space 1000 m circular buffer were related to greater standing long jump distance, accessible green space (500 m and 1000 m circular buffer) was related to shorter jump distance (see Fig. 2). However, for accessible green space (250–1000 m circular buffer; 1000 m street-network buffer), interaction analysis revealed that age moderated the association, indicating that the negative relationship between accessible green space and standing long jump distance only occurred for adolescents, but not for children (see Additional file 1: Fig. S3).

### Natural environment and mental health problems

Accessible green space street-network buffers (3000-5000 m) were positively related to the SDQ score, indicating greater mental health problems with more green space (see Fig. 2). Interaction analysis revealed distinct associations depending on participants’ characteristics (see Additional file 1: Figs. S4, S5). For socio-economic status, the results showed that both nature and green space circular (500 m) and street-network-buffers (1000 m) were related to less mental health problems for children and adolescents with low socio-economic status. For children and adolescents with high socio-economic status, less mental health problems were consistently observed across the nature and green space street-network buffers (1000–5000 m). However, the accessible greenspace street-network buffer (3000 m) was associated with greater mental health problems in children and adolescents with low socio-economic status.

Regarding age, interaction results revealed that all accessible greenspace circular and street-network buffers (except for the 100 m circular buffer) were associated with greater mental health problems in adolescents, but not in children.

## Discussion

The goal of this study was to explore variations in the relationship between natural environments and different health outcomes in children and adolescents. Our study showed heterogenous results depending on buffer size and buffer type and expands previous research by demonstrating that this variation also depends on the nature definition, the health outcome under investigation, and the sample’s characteristics.

More specifically, some buffer sizes of the natural environment and green space showed statistically significant negative relationships with physical activity behavior. However, when only accessible green space (excluding agricultural areas) was considered, no relationship emerged. Further, the relationships only emerged for natural environments and green space with weekday physical activity, while there was no association with weekend day physical activity observed. Also, the result differed by socio-economic status. While we do not have a clear explanation for these results, a potential reason may be that more exposure to natural environments reflects less access to other environmental features that are related to children’s and adolescent’s physical activity, such as short distances to leisure and sports facilities, mixed land use, or infrastructure for walking and cycling [13, 59, 66]. Additionally, during childhood and adolescents, other aspects, such as peer- and parental social support [59], may be more important for physical activity engagement than residential green space. These heterogenous results for physical activity based on green space definition and buffer sizes are in line with a previous study in adults that investigated green space type (NDVI vs. green space based on land-use data) and various circular buffer sizes (ranging from 100 to 3000 m) in relation to outdoor physical activity [21]. Similar to the variations observed in this study, Klompmaker et al. found that compared to people in the lowest quintile of greenspace exposure, people in higher quintiles (representing people with more green space exposure) had a lower likelihood of being at least 150 min per week active outdoors for the 100 m buffer and 500–3000 m buffers, but not for the 300 m buffer [21].

For long jump distance, statistically significant positive associations emerged for nature and green space (1000 m circular buffer), whereas a statistically significant negative relationship occurred for the same buffer size and type when looking at accessible green space. We were unable to find previous studies that investigated green space types and buffers in relation to muscular fitness, however, a systematic review investigating variations of buffer size in relation to physical health outcomes (e.g., obesity, cardiovascular disease) in children and adults also found that the observed relationship was dependent on the buffer size, with the likelihood of greenness being associated with physical health being the highest for buffers between 500 and 999 m when using home addresses as buffer centers [38]. The authors argued that this indicates that individuals with high green density in the broader neighborhood have better physical health than individuals with high green density in their immediate surroundings but low green density in the broader neighborhood [38].

For mental health problems, two accessible green space street-network buffers were statistically significant related to greater mental health problems, whereas there were no statistically significant relationships with any of the other buffers when looking at the complete sample, which is in line with previous inconsistent results summarized in a systematic review due to a high variability in the metrics used to quantify natural environments [37].

**Fig. 3:**
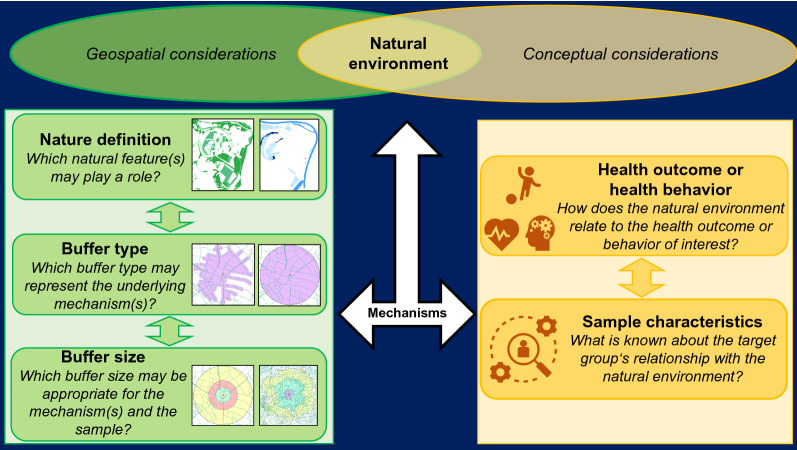
Conceptual framework integrating geospatial and conceptual considerations for developing and choosing nature assessment in health research studies

These heterogenous results demonstrate that it is vital to consider the nature definition as well as buffer size and buffer type carefully when configuring the natural environment for one’s study. In the following, we outline some guiding questions as a framework that may be used when deciding on how to choose the appropriate nature variable in one’s study. We argue that it is necessary to integrate both geospatial and conceptual considerations when configuring the natural environment for one’s study. A conceptual framework that may be used to guide one’s decisions on the configuration is presented in Fig. 3 and discussed in the following.

### (a) Nature definition

In our study, we assessed nature in three different ways: (a) nature, including both green and blue space, hence representing any land-use area that could be counted as natural environment; (b) green space, which excluded water-based areas, and (c) accessible green space, excluding agricultural areas as they are often not accessible to the public. Overall, associations varied across nature definitions and health outcomes or health behavior.

Hence, we argue that it is important to consider which geospatial definition of nature aligns best with the conceptual assumptions and potential mechanisms linking nature and the health outcome under investigation. GIS provide multiple options to operationalize natural environments. For example, for green space, common methods include remote sensing from satellite images to detect the density of green vegetation, resulting in the normalized difference vegetation index (NDVI) [67], or using national land use databases [68]. However, green space operationalized as NDVI or land use may represent different underlying mechanisms: If one assumes that the mechanism underlying the green space—health relationship is related to vegetation density, NDVI could be the most appropriate measure. In contrast, if one assumes that the mechanism underlying the green space—health relationship is driven by the use of green space for activity or recreation, operationalizing green space via land use data is probably more appropriate. It has been previously demonstrated that using the NDVI index or land cover and land use data yields heterogenous results regarding the relationship with overweight/obesity and physical activity [21].

Beyond the geographical definition of the natural environment, specific mechanisms that may link nature and the health outcome under investigation should be considered when deciding which types and features of the natural environment should be included. For example, decreased air pollution has been suggested as one important mechanism that mediates the relationship between nature exposure and mental well-being [9]. When air pollution is expected to be a central mechanism, it may be appropriate to exclude water-based areas as their mitigating effect on air pollution is considered much weaker compared to green space [69]. However, when considering social cohesion and interactions as a potential mechanism, the inclusion of both green space and blue space may be appropriate [10, 69]. If the quality of the natural environment is assumed to play a role, aspects like biodiversity or amenities may be appropriate to consider [70]. This can be expanded to considering the mechanisms in the context of the sample’s characteristics: for example, a recent review outlined potential mechanisms of green space interventions for mental health considering contextual conditions and people’s characteristics, concluding that mechanisms such as escaping from everyday life and being alone in natural environments may be specifically relevant for psychologically vulnerable people, while mental health benefits from natural environments for women may be mitigated due to a higher aversion to the outdoors [71].

Thus, we argue that it is vital to conceptualize the underlying mechanism between the natural environment and the health outcome while considering the unique characteristics of the sample that may impact those mechanisms prior to the analysis.

### (b) Buffer type

In our study, we used two different individualized buffer types, including circular buffers and street-network buffers, which showed distinct and partially inverse relationships with the health outcome under investigation. Other typical buffer types include administrative boundaries (e.g., census tracts) or grid cells [18, 19]. As outlined in the introduction, the choice of buffer type and buffer size determines the spatial configuration, which often has a large influence on the relationship under investigation, known as the MAUP [16]. To minimize this problem, several approaches exist, e.g., the use of disaggregated data [18]. Similar to the nature definition, considering the outcome under investigation and the potential underlying mechanism may be useful when deciding on the buffer type. For example, when examining the natural environment in relation to physical activity, one may assume that accessibility to the natural environment, such as a walking path along a river, is vital. This can be captured when using a network buffer, but less so when using circular buffers [72]. In contrast, when investigating mental health outcomes, visual exposure may be more important than accessibility. Hence, a circular buffer may be appropriate [73]. Furthermore, the buffer type may also depend on the study’s objective. For example, if the goal is to inform policymakers about green space interventions within communities, administrative community boundaries may be appropriate in combination with statistical methods that account for the clustering of the data within the communities, such as multilevel modeling [18]. To create individual-level buffers, another, more advanced approach is to assess one’s actual activity space using ambulatory assessment methods, such as logging devices that record one’s whereabouts using global positioning systems, thereby allowing to match environmental characteristics and time with high spatial and temporal resolution [74]. This approach can be extended to other sensors, such as accelerometers [75] and e-diaries [76] to assess behavior and psychological constructs in the context of natural environments. The value of such approaches becomes clear when considering the uncertain geographic context problem, referring to how much the spatial area used for a study deviates from the true causal geographically relevant context [77]. For example, a study with US adolescents showed that half of the participants spent 92% of their outdoor time outside their census tract area of residence [78]. In this case, assessing the neighborhood via the census tract would not represent a relevant geographical context. Another study showed that the closest urban green space was not the one that was used most, with the use being dependent on the features and facilities of the particular green space [60]. Ambulatory assessment methods that capture one’s actual activity space, such as combining accelerometry use with geolocation tracking, can overcome such problems as they provide utilization information, such as time spent in green space and physical activity levels whilst being exposed to green space [79, 80]. However, caution about causal inferences is also necessary with ambulatory assessment approaches as new challenges, such as the selective mobility bias, with individuals actively seeking places for specific purposes, such as a park for physical activity or specific restaurants based on their food preferences, may arise [81, 82].

### (c) Buffer size

In our study, we used buffer sizes from 100 to 1000 m for circular buffers and 1000 m to 5000 m for the street-network buffers based on previous health research studies with children and adolescents [15]. For the overall sample, the 500 m to 5000 m buffer distances were the ones that mainly played a role, with distinct relationships depending on buffer type, nature definition, and health outcome, which is an inherent problem addressed in the MAUP [16, 18]. When looking at the results of the interaction analysis by gender, age, and socio-economic status, we observed that different buffer sizes were relevant for different subgroups and that those relationships were again distinct. For example, for children and adolescents with high socio-economic status, circular buffer sizes ranging from 100 to 500 m were consistently related to less MVPA compared to youth with medium socio-economic status. For mental health problems, street-network buffers of 1000 m to 5000 m for nature and green space were related to greater mental health problems in children and adolescents with high socio-economic status compared to children and adolescents with medium socio-economic status. However, in children and adolescents with low socio-economic status, less mental health problems were observed for the 1000 m circular and 3000 m street-network accessible green space buffers, but greater mental health problems for the 500 m circular and 1000 m street-network accessible green space buffers compared to youth with medium socio-economic status.

This makes the scale choice of the buffer size as an integral part of the spatial configuration as the nature definition and buffer type. To address this issue, it has been suggested, amongst others, to conduct a sensitivity analysis using different buffer sizes to explore the magnitude of the MAUP in one’s data [16]. While a sensitivity analysis allow to investigate the scope of the MAUP, we argue that the primary buffer size should be determined a-priori based on both geospatial and conceptual considerations that link the natural environment to the health outcome under investigation. For example, a previous study investigating momentary associations between urban green space and mood used 100 m circular buffers around participants’ geolocations for assessing momentary green space based on the assumption that mood benefits would be the result of visual green space exposure and considering that surrounding buildings in the city allow only a limited view [73]. In addition, this study supported their buffer size choice with a quantitative analysis that estimated the visual range in the city [73]. On a more general level, conceptually, smaller buffer sizes seem to provide better assessments than larger buffer sizes when using ambulatory assessment approaches for geolocation data in health research [83].

Furthermore, our analysis revealed that the choice of scale should not only be considered in the context of the nature definition, buffer type, and health outcome, but also the context of the sample. Especially in large datasets with heterogenous participants, this may require specifying buffer sizes for sub-groups. Here again, conceptual and geospatial considerations should be integrated. For example, it was shown that socio-economically disadvantaged groups experience less green space access and quality [84, 85], which also mirrors in differential use of green space for physical activity purposes of people with different income levels [86]. In contrast, for mental health, another study showed that green space had a stronger relation to a reduced likelihood of depressive symptoms in pregnant women with lower education [87]. Gender- and age-based differences occurred in children and adolescents, with a longer distance to parks being related to less physical activity of 6-to-11 year-old boys and girls, but only to less physical activity of male adolescents (12 to 17 years), whereas there emerged no relationship for female adolescents [88]. These empirical findings should be conceptually considered when deciding on the buffer size for one’s study and how this may impact the underlying mechanisms linking the natural environment to health outcomes and behavior.

### Strengths and limitations

Our study has several limitations. First, the cross-sectional character of our dataset does not allow causal inferences. Second, we used federal land cover and land use data that does not consider private natural environments, such as gardens. Third, while we do have GIS-based information regarding residential natural environments, we do not have information regarding the utilization of the natural environment through children and their parents. Finally, for physical activity, we only had a sub-sample of participants who agreed to wear an accelerometer for 1 week, potentially inducing selection bias. Comparing accelerometer participants (= sub-sample) with the sample that did not agree to wear an accelerometer or had invalid accelerometer data, we observed statistically significant differences regarding socio-economic status (participants with low socio-economic status being less likely to be part of the accelerometer sub-sample), BMI (participants with overweight/obesity being less likely to be part of the sub-sample), and exposure to the natural environment, with participants of the sub-sample having statistically significant more nature exposure for the majority of the nature and buffer types and buffer sizes. However, when looking at effect sizes, these effects were small. No statistically significant differences were observed regarding age and gender.

Nonetheless, we would like to highlight that this is one of the first studies that investigated variations in the association between natural environments and health in a broad sample of children and adolescents, considering various geospatial configurations, health outcomes and behavior, and sample characteristics.

## Conclusion

The goal of this study was to demonstrate varying relationships between natural environments and selected examples of health outcomes and behavior based on nature definition, buffer type, and buffer size while accounting for specific sample characteristics. As there is no consensus on the geospatial configuration of the natural environment in health research, our second aim was to provide a framework and guiding questions that may facilitate the spatial configuration of the natural environment in future studies. We argue that future studies should integrate geospatial considerations (nature definition, buffer type, and buffer size) with conceptual considerations (health outcome and behavior, sample characteristics), taking into account potential mechanisms, to provide better reasoning and understanding of the relationship between natural environments and health (behavior).

## Supplementary Information

**Additional file 1: Text S1.** Equations of the linear regression models. **Table S1.** Descriptive results of the whole study sample regarding socio-demographic information, weight status, and outcome variables (N = 2843). **Table S2.** Results of the multiple linear regression models predicting moderate-to-vigorous physical activity. **Table S3.** Results of the multiple linear regression models predicting long jump (centimeters). **Table S4.** Results of the multiple linear regression models predicting mental health. **Table S5.** Selected models including the reporting of co-variates. **Figure S1.** Variation regarding the relationship between nature indices, buffer types, and buffer distances for moderate-to-vigorous physical activity (MVPA) across youth with low and high socio-economic status compared to youth with medium socio-economic status (reference category). **Figure S2.** Variation regarding the relationship between nature indices, buffer types, and buffer distances for moderate-to-vigorous physical activity (MVPA) at the weekend and during the week. **Figure S3.** Variations regarding the relationship between nature indices, buffer types, and buffer distances across age for standing long jump distance. **Figure S5.** Variation regarding the relationship between nature indices, buffer types, and buffer distances across age regarding SDQ score.

## Data Availability

The datasets generated and analyzed during the current study are not publicly available due to the strict ethical standards required by the Federal Office for the Protection of Data with which study investigators are obliged to comply but are available from the corresponding author upon reasonable request.
