# Supplementary material for: The geospatial and conceptual configuration of the natural environment impacts the association with health outcomes and behavior in children and adolescents
Source: Int J Health Geogr. 2022 Aug 11;21:9. doi: 10.1186/s12942-022-00309-0 (PMC9366780; doi:10.1186/s12942-022-00309-0)
Supplement: Supplementary file 1 — Additional file 1: Text S1. Equations of the linear regression models. Table S1. Descriptive results of the whole study sample regarding socio-demographic information, weight status, and outcome variables (N = 2843). Table S2. Results of the multiple linear regression models predicting moderate-to-vigorous physical activity. Table S3. Results of the multiple linear regression models predicting long jump (centimeters). Table S4. Results of the multiple linear regression models predicting mental health. Table S5. Selected models including the reporting of co-variates. Figure S1. Variation regarding the relationship between nature indices, buffer types, and buffer distances for moderate-to-vigorous physical activity (MVPA) across youth with low and high socio-economic status compared to youth with medium socio-economic status (reference category). Figure S2. Variation regarding the relationship between nature indices, buffer types, and buffer distances for moderate-to-vigorous physical activity (MVPA) at the weekend and during the week. Figure S3. Variations regarding the relationship between nature indices, buffer types, and buffer distances across age for standing long jump distance. Figure S5. Variation regarding the relationship between nature indices, buffer types, and buffer distances across age regarding SDQ score. [file 12942_2022_309_MOESM1_ESM.docx]

**Additional File 1**

**The Geospatial and Conceptual Configuration of the Natural Environment Impacts the Association with Health Outcomes and Behavior in Children and Adolescents**

**S1. Equations of the linear regression models**

*Moderate-to-vigorous physical activity (MVPA)*

$${Y(\boldsymbol{MVPA})}_{j}=\beta_{00}+\beta_{01}*{nature\_index}_{j}+ \beta_{02}*{age}_{j}+\beta_{03}*{sex}_{j}+\beta_{04}*{BMI}_{j}+\beta_{05}*{socio-economic status}_{j}+r_{j}$$

*Standing long jump distance*

$${Y(\boldsymbol{Standing long jump distance})}_{j}=\beta_{00}+\beta_{01}*{nature\_index}_{j}+ \beta_{02}*{age}_{j}+\beta_{03}*{sex}_{j}+\beta_{04}*{BMI}_{j}+\beta_{05}*{socio-economic status}_{j}+r_{j}$$

*Mental health assessed via the Strength and Difficulties Questionnaire (SDQ) score*

$${Y(\boldsymbol{SDQ score})}_{j}=\beta_{00}+\beta_{01}*{nature\_index}_{j}+ \beta_{02}*{age}_{j}+\beta_{03}*{sex}_{j}+\beta_{04}*{BMI}_{j}+\beta_{05}*{socio-economic status}_{j}+r_{j}$$

*Example interaction analysis for nature index by socio-economic status*

*Moderate-to-vigorous physical activity (MVPA)*

$${Y(\boldsymbol{MVPA})}_{j}=\beta_{00}+\beta_{01}*{natur{e\_index}_{j}}+ \beta_{02}*{age}_{j}+\beta_{03}*{sex}_{j}+\beta_{04}*{BMI}_{j}+\beta_{05}*{socio-economic status}_{j}+ \beta_{06}*{{nature\_index}_{j} * socio-economic status}_{j}+r_{j}$$

*Table S1. Descriptive results of the whole study sample regarding socio-demographic information, weight status, and outcome variables (N = 2,843)*

|  | **MVPA**  **(N=923)** | **Standing long jump**  **(N = 2,493)** | **Mental health problems (N = 2,341)** |
| --- | --- | --- | --- |
| **Socio-demographic information**  **and weight status** |  |  |  |
| Age in years (mean, SD) | 11.19 (3.34) | 10.37 (3.96) | 10.42 (3.94) |
| Gender |  |  |  |
| Boys | 461 (49.9%) | 1294 (51.9%) | 1213 (51.8%) |
| Girls | 462 (50.1%) | 1199 (48.1%) | 1128 (48.2%) |
| BMI based on IOTF cutpoints |  |  |  |
| Underweight | 86 (9.3%) | 241 (9.7%) | 230 (9.8%) |
| Normal weight | 706 (76.5%) | 1887 (75.7%) | 1773 (75.7%) |
| Overweight | 111 (12.0%) | 284 (11.4%) | 268 (11.4%) |
| Obese | 20 (2.2%) | 81 (3.2%) | 70 (3.0%) |
| Socio-economic status |  |  |  |
| Low | 164 (17.8%) | 489 (19.6%) | 447 (19.1%) |
| Medium | 532 (57.6%) | 1434 (57.5%) | 1360 (58.1%) |
| High | 227 (24.6%) | 570 (22.9%) | 534 (22.8%) |
|  |  |  |  |
| **Circular buffer % (mean, SD)** |  |  |  |
| Nature 100m | 17.71 (21.12) | 16.79 (20.57) | 17.02 (20.57) |
| Nature 250m | 33.20 (23.98) | 30.96 (23.22) | 31.33 (23.25) |
| Nature 500m | 46.46 (24.55) | 43.59 (24.00) | 43.97 (24.00) |
| Nature 1000m | 59.27 (24.02) | 56.63 (23.49) | 57.05 (23.37) |
| Greenspace 100m | 17.42 (20.99) | 16.51 (20.43) | 16.75 (20.42) |
| Greenspace 250m | 32.53 (23.86) | 30.31 (23.10) | 30.67 (23.13) |
| Greenspace 500m | 45.30 (24.70) | 42.42 (23.99) | 42.81 (23.98) |
| Greenspace 1000m | 57.78 (24.48) | 54.96 (23.81) | 55.40 (23.70) |
| Accessible greenspace 100m | 6.053 (12.37) | 5.990 (12.03) | 6.085 (12.20) |
| Accessible greenspace 250m | 10.54 (13.12) | 10.56 (12.48) | 10.58 (12.56) |
| Accessible greenspace 500m | 14.45 (13.89) | 14.18 (12.80) | 14.16 (12.87) |
| Accessible greenspace 1000m | 18.54 (14.00) | 18.09 (13.13) | 18.11 (13.19) |
|  |  |  |  |
| **Street-network buffer % (mean, SD)** |  |  |  |
| Nature 1000m | 42.50 (23.19) | 39.65 (22.73) | 40.04 (22.63) |
| Nature 3000m | 64.71 (23.21) | 62.27 (22.52) | 62.61 (22.48) |
| Nature 5000m | 71.49 (20.91) | 69.59 (20.24) | 69.83 (20.20) |
| Greenspace 1000m | 41.90 (23.29) | 39.00 (22.77) | 39.40 (22.68) |
| Greenspace 3000m | 63.90 (23.28) | 61.11 (22.81) | 61.43 (22.78) |
| Greenspace 5000m | 70.63 (21.24) | 68.56 (20.57) | 68.81 (20.51) |
| Accessible greenspace 1000m | 13.89 (12.51) | 13.49 (11.75) | 13.54 (11.83) |
| Accessible greenspace 3000m | 23.18 (14.34) | 22.34 (13.73) | 22.46 (13.82) |
| Accessible greenspace 5000m | 26.14 (14.59) | 25.14 (13.91) | 25.27 (13.97) |
|  |  |  |  |
| **Outcome variable (mean, SD)** |  |  |  |
| MVPA (minutes/day) | 53.18 (23.50) |  |  |
| Standing long jump distance (centimeters) |  | 136.61 (37.49) |  |
| Mental health problems (SDQ score) |  |  | 9.49 (5.00) |

*Table S2. Results of the multiple linear regression models predicting moderate-to-vigorous physical activity.*

| *Nature predictor in the model* | *Intercept* | *SE Intercept* | *B Nature predictor* | *SE B Nature predictor* | *p* | *Adj. R^2^* |
| --- | --- | --- | --- | --- | --- | --- |
| **Circular buffer** |  |  |  |  |  |  |
| Nature100m | 59.52 | 1.14 | 0.00 | 0.00 | 0.968 | 0.287 |
| Nature250m | 59.56 | 1.14 | -0.04 | 0.03 | 0.165 | 0.289 |
| Nature500m | 59.58 | 1.14 | **-0.06** | 0.03 | 0.023 | 0.291 |
| Nature1000m | 59.67 | 1.14 | **-0.07** | 0.03 | 0.009 | 0.293 |
| Greenspace100m | 59.52 | 1.14 | 0.00 | 0.00 | 0.967 | 0.287 |
| Greenspace250m | 59.56 | 1.14 | -0.04 | 0.03 | 0.177 | 0.289 |
| Greenspace500m | 59.56 | 1.14 | **-0.06** | 0.03 | 0.032 | 0.291 |
| Greenspace1000m | 59.63 | 1.14 | **-0.07** | 0.03 | 0.015 | 0.292 |
| AccessibleGreen100m | 59.55 | 1.14 | 0.04 | 0.05 | 0.406 | 0.288 |
| AccessibleGreen250m | 59.58 | 1.14 | 0.07 | 0.05 | 0.160 | 0.289 |
| AccessibleGreen500m | 59.57 | 1.14 | 0.09 | 0.05 | 0.064 | 0.290 |
| AccessibleGreen1000m | 59.57 | 1.14 | 0.08 | 0.05 | 0.088 | 0.290 |
| **Street-network buffers** |  |  |  |  |  |  |
| Nature1000m | 59.57 | 1.14 | -0.04 | 0.03 | 0.130 | 0.289 |
| Nature3000m | 59.64 | 1.14 | **-0.06** | 0.03 | 0.046 | 0.291 |
| Nature5000m | 59.66 | 1.14 | **-0.08** | 0.03 | 0.011 | 0.293 |
| Greenspace1000m | 59.57 | 1.14 | -0.04 | 0.03 | 0.147 | 0.289 |
| Greenspace3000m | 59.64 | 1.14 | -0.03 | 0.03 | 0.263 | 0.288 |
| Greenspace5000m | 59.65 | 1.14 | **-0.08** | 0.03 | 0.012 | 0.292 |
| AccessibleGreen1000m | 59.59 | 1.14 | 0.06 | 0.05 | 0.254 | 0.288 |
| AccessibleGreen3000m | 59.56 | 1.14 | 0.03 | 0.05 | 0.546 | 0.288 |
| AccessibleGreen5000m | 59.50 | 1.14 | 0.02 | 0.04 | 0.720 | 0.288 |

*Please note: All models were controlled for age (centered on the sample’s mean), gender (ref. category: males), socio-economic status (ref. category: medium), and BMI (ref. category: normal weight.* ***Bolded*** *values indicate p < 0.05*

*Table S3. Results of the multiple linear regression models predicting long jump (centimeters).*

| *Nature predictor in the model* | *Intercept* | *SE Intercept* | *B Nature predictor* | *SE B Nature predictor* | *p* | *Adj. R^2^* |
| --- | --- | --- | --- | --- | --- | --- |
| **Circular buffer** |  |  |  |  |  |  |
| Nature100m | 145.77 | 0.75 | 0.00 | 0.02 | 0.893 | 0.669 |
| Nature250m | 145.77 | 0.75 | 0.02 | 0.02 | 0.329 | 0.669 |
| Nature500m | 145.76 | 0.75 | 0.03 | 0.02 | 0.078 | 0.669 |
| Nature1000m | 145.73 | 0.75 | **0.04** | 0.02 | 0.031 | 0.669 |
| Greenspace100m | 145.77 | 0.75 | 0.00 | 0.02 | 0.925 | 0.669 |
| Greenspace250m | 145.76 | 0.75 | 0.02 | 0.02 | 0.356 | 0.669 |
| Greenspace500m | 145.76 | 0.75 | 0.03 | 0.02 | 0.075 | 0.669 |
| Greenspace1000m | 145.74 | 0.75 | **0.04** | 0.02 | 0.024 | 0.669 |
| AccessibleGreen100m | 145.72 | 0.75 | -0.04 | 0.04 | 0.228 | 0.669 |
| AccessibleGreen250m | 145.71 | 0.75 | -0.06 | 0.03 | 0.069 | 0.669 |
| AccessibleGreen500m | 145.73 | 0.75 | **-0.08** | 0.03 | 0.026 | 0.669 |
| AccessibleGreen1000m | 145.75 | 0.75 | **-0.09** | 0.03 | 0.008 | 0.670 |
| **Street-network buffers** |  |  |  |  |  | 0.669 |
| Nature1000m | 145.73 | 0.75 | 0.04 | 0.02 | 0.067 | 0.669 |
| Nature3000m | 145.72 | 0.75 | 0.03 | 0.02 | 0.099 | 0.669 |
| Nature5000m | 145.73 | 0.75 | 0.04 | 0.02 | 0.104 | 0.669 |
| Greenspace1000m | 145.74 | 0.75 | 0.04 | 0.02 | 0.063 | 0.669 |
| Greenspace3000m | 145.75 | 0.75 | 0.02 | 0.02 | 0.308 | 0.669 |
| Greenspace5000m | 145.73 | 0.75 | 0.03 | 0.02 | 0.100 | 0.669 |
| AccessibleGreen1000m | 145.75 | 0.75 | -0.06 | 0.04 | 0.127 | 0.669 |
| AccessibleGreen3000m | 145.73 | 0.75 | 0.03 | 0.03 | 0.420 | 0.669 |
| AccessibleGreen5000m | 145.73 | 0.75 | -0.01 | 0.03 | 0.808 | 0.669 |

*Please note: All models were controlled for age (centered on the sample’s mean), gender (ref. category: males), socio-economic status (ref. category: medium), and BMI (ref. category: normal weight.* ***Bolded*** *values indicate p < 0.05*

*Table S4. Results of the multiple linear regression models predicting mental health.*

| *Nature predictor in the model* | *Intercept* | *SE Intercept* | *B Nature predictor* | *SE B Nature predictor* | *p* | *Adj. R^2^* |
| --- | --- | --- | --- | --- | --- | --- |
| **Circular buffer** |  |  |  |  |  |  |
| Nature100m | 9.46 | 0.17 | -0.01 | 0.00 | 0.073 | 0.043 |
| Nature250m | 9.46 | 0.17 | 0.00 | 0.00 | 0.876 | 0.042 |
| Nature500m | 9.46 | 0.18 | 0.00 | 0.00 | 0.805 | 0.042 |
| Nature1000m | 9.47 | 0.18 | 0.00 | 0.00 | 0.266 | 0.042 |
| Greenspace100m | 9.46 | 0.18 | -0.01 | 0.00 | 0.074 | 0.043 |
| Greenspace250m | 9.46 | 0.18 | 0.00 | 0.00 | 0.857 | 0.042 |
| Greenspace500m | 9.46 | 0.18 | -0.02 | 0.00 | 0.593 | 0.042 |
| Greenspace1000m | 9.47 | 0.18 | -0.01 | 0.00 | 0.119 | 0.043 |
| AccessibleGreen100m | 9.46 | 0.18 | -0.01 | 0.01 | 0.512 | 0.042 |
| AccessibleGreen250m | 9.47 | 0.18 | 0.01 | 0.01 | 0.201 | 0.042 |
| AccessibleGreen500m | 9.48 | 0.18 | 0.01 | 0.01 | 0.156 | 0.042 |
| AccessibleGreen1000m | 9.46 | 0.18 | 0.01 | 0.01 | 0.095 | 0.043 |
| **Street-network buffers** |  |  |  |  |  |  |
| Nature1000m | 9.47 | 0.18 | 0.00 | 0.00 | 0.398 | 0.042 |
| Nature3000m | 9.47 | 0.18 | -0.01 | 0.00 | 0.166 | 0.042 |
| Nature5000m | 9.47 | 0.18 | -0.01 | 0.01 | 0.289 | 0.042 |
| Greenspace1000m | 9.47 | 0.18 | 0.00 | 0.00 | 0.394 | 0.042 |
| Greenspace3000m | 9.47 | 0.18 | -0.01 | 0.00 | 0.154 | 0.042 |
| Greenspace5000m | 9.47 | 0.18 | -0.01 | 0.00 | 0.280 | 0.042 |
| AccessibleGreen1000m | 9.47 | 0.18 | 0.01 | 0.01 | 0.101 | 0.043 |
| AccessibleGreen3000m | 9.46 | 0.17 | **0.02** | 0.01 | 0.015 | 0.044 |
| AccessibleGreen5000m | 9.47 | 0.17 | **0.03** | 0.01 | <0.001 | 0.047 |

*Please note: All models were controlled for age (centered on the sample’s mean), gender (ref. category: males), socio-economic status (ref. category: medium), and BMI (ref. category: normal weight.* ***Bolded*** *values indicate p < 0.05*

Table S5. Selected models including the reporting of co-variates.

|  | **MVPA**  **(N = 923)** | | | | | **SDQ score**  **(N = 2,341)** | | | | | **Long jump distance**  **(N = 2,493)** | | | | |
| --- | --- | --- | --- | --- | --- | --- | --- | --- | --- | --- | --- | --- | --- | --- | --- |
| *Predictors* | *B* | *95%CI* | *SE* | *β* | *p* | *B* | *95%CI* | *SE* | *β* | *p* | *B* | *95%CI* | *SE* | *β* | *p* |
| (Intercept) | 59.67 | 57.43;61.90 | 1.14 | 0.08 | **<0.001** | 9.47 | 9.12;9.81 | 0.18 | -0.05 | **<0.001** | 145.74 | 144.28;147.21 | 0.75 | 0.06 | **<0.001** |
| Nature 1000m  circular buffer | -0.07 | -0.13;-0.02 | 0.03 | -0.07 | **0.009** | -0.00 | -0.01;0.00 | 0.00 | -0.02 | 0.266 | 0.04 | 0.00;0.08 | 0.02 | 0.02 | **0.031** |
| Age | -3.18 | -3.57;-2.79 | 0.20 | -0.45 | **<0.001** | 0.09 | 0.04;0.14 | 0.03 | 0.07 | **<0.001** | 7.54 | 7.32;7.75 | 0.11 | 0.80 | **<0.001** |
| Socio-economic status  (ref. medium) |  |  |  |  |  |  |  |  |  |  |  |  |  |  |  |
| Low | 0.69 | -2.79;4.18 | 1.78 | 0.03 | 0.696 | 1.42 | 0.90;1.95 | 0.27 | 0.28 | **<0.001** | -4.99 | -7.22;-2.75 | 1.14 | -0.13 | **<0.001** |
| High | -0.85 | -3.96;2.27 | 1.59 | -0.04 | 0.593 | -0.86 | -1.35;-0.36 | 0.25 | -0.17 | **0.001** | 4.17 | 2.06;6.28 | 1.07 | 0.11 | **<0.001** |
| Gender (ref. boys) | -9.42 | -11.99;-6.85 | 1.31 | -0.20 | **<0.001** | -0.48 | -0.87;-0.08 | 0.20 | -0.05 | **0.019** | -12.85 | -14.54;-11.15 | 0.87 | -0.17 | **<0.001** |
| IOTF  (ref. normal weight) |  |  |  |  |  |  |  |  |  |  |  |  |  |  |  |
| Underweight | -4.84 | -9.28;-0.40 | 2.26 | -0.21 | **0.032** | 0.17 | -0.50;0.85 | 0.34 | 0.03 | 0.617 | -0.72 | -3.62;2.17 | 1.48 | -0.02 | 0.624 |
| Overweight | -8.85 | -12.83;-4.86 | 2.03 | -0.38 | **<0.001** | 0.76 | 0.13;1.40 | 0.32 | 0.15 | **0.018** | -11.82 | -14.53;-9.11 | 1.38 | -0.32 | **<0.001** |
| Obese | -12.47 | -21.31;-3.63 | 4.50 | -0.53 | **0.006** | 2.70 | 1.52;3.89 | 0.60 | 0.54 | **<0.001** | -25.37 | -30.22;-20.53 | 2.47 | -0.68 | **<0.001** |

*Please note: The relationship between the co-variates and health outcomes remained stable across all models with varying nature operationalizations, buffer types, and buffer sizes.*

Figure S1*. Variation regarding the relationship between nature indices, buffer types, and buffer distances for moderate-to-vigorous physical activity (MVPA) across youth with low and high socio-economic status compared to youth with medium socio-economic status (reference category).*


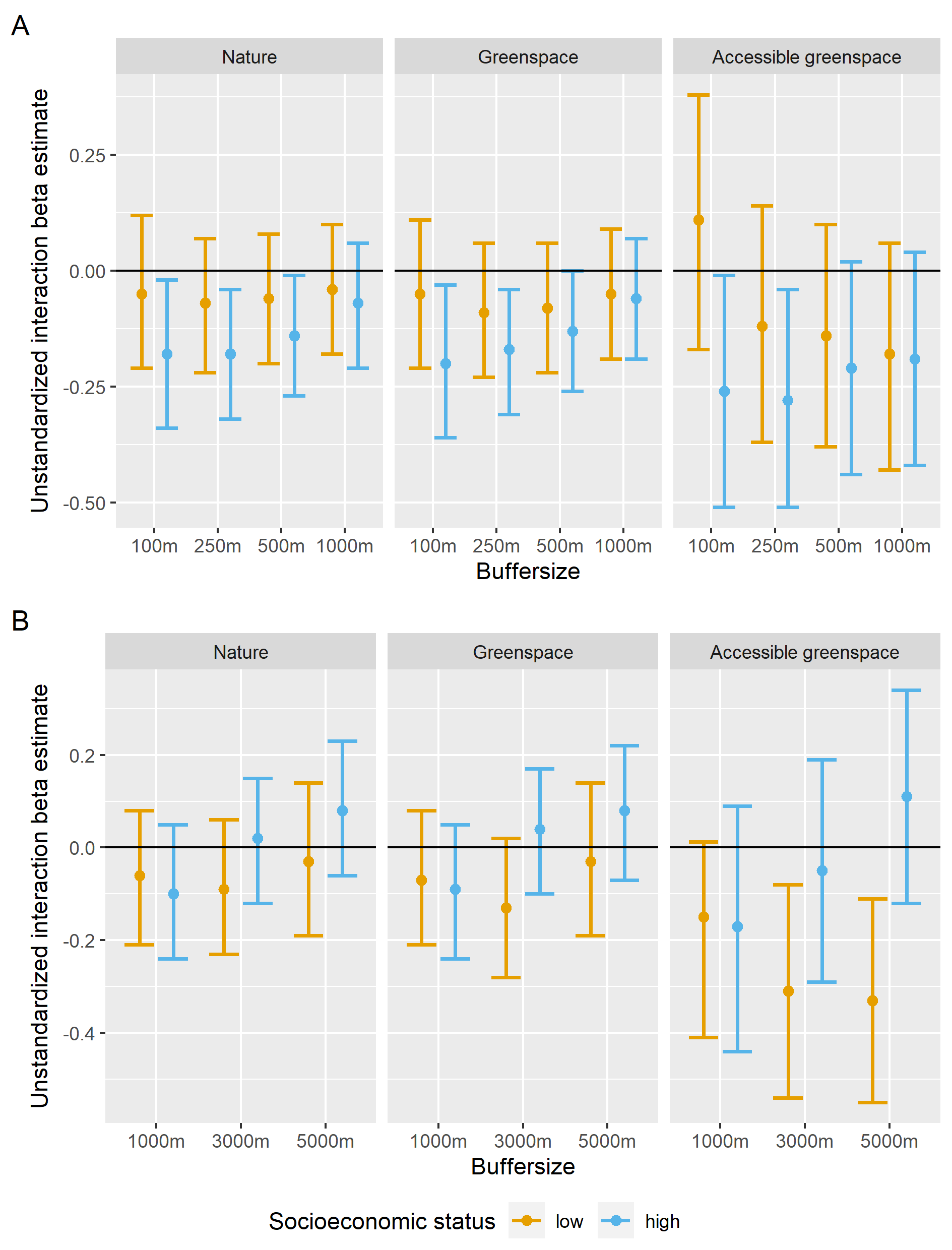


*Panel A: Unstandardized beta estimates for circular buffers. Panel B: Unstandardized beta estimates for street-network buffers Sample size: N = 923. Error bars represent 95% confidence intervals. All models were adjusted for age, gender, BMI, and socio-economic status.*

Figure S2. *Variation regarding the relationship between nature indices, buffer types, and buffer distances for moderate-to-vigorous physical activity (MVPA) at the weekend and during the week.*


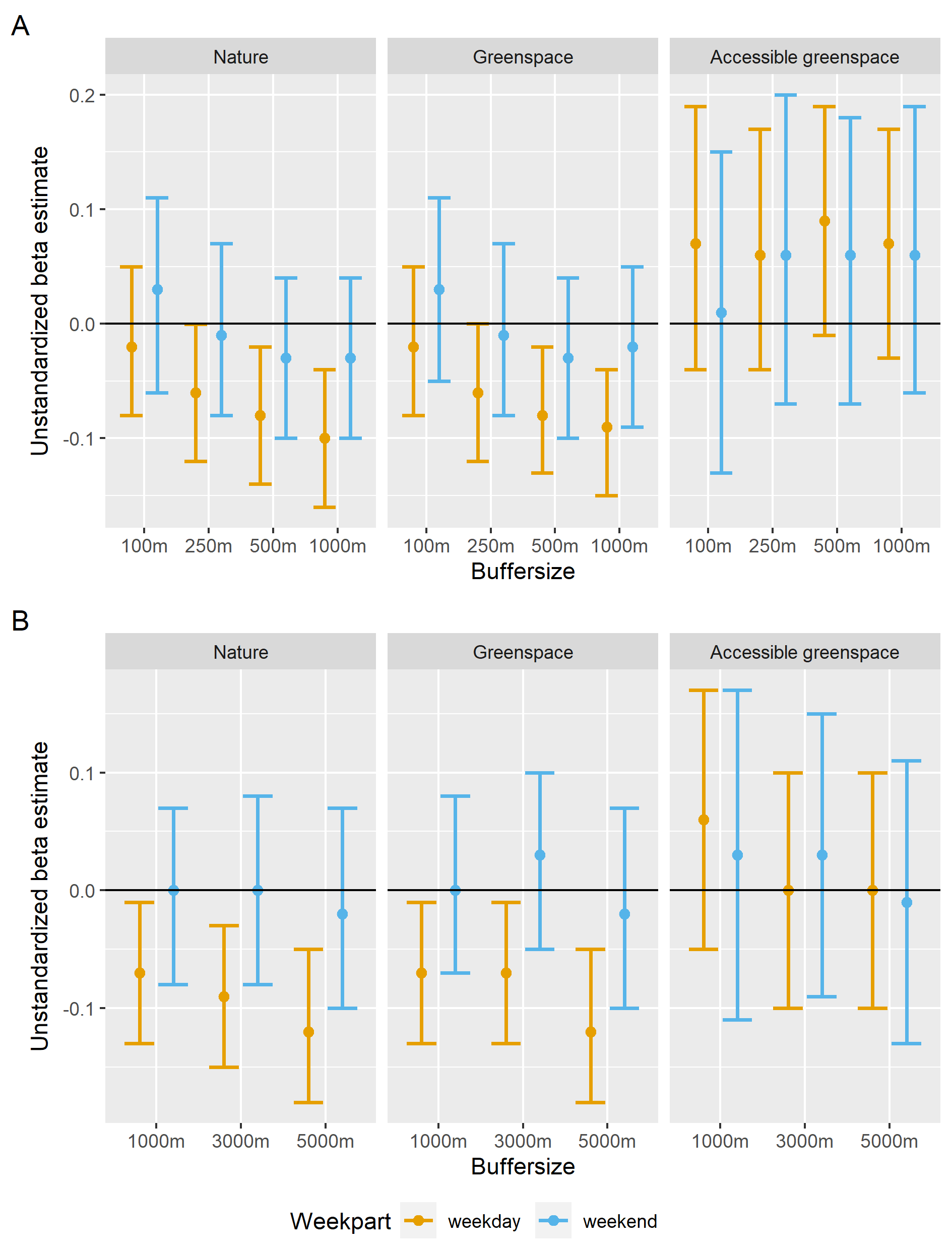


*Panel A: Unstandardized interaction beta estimates for circular buffers. Panel B: Unstandardized interaction beta estimates for street-network buffers. Sample size: N = 923. Error bars represent 95% confidence intervals. All models were adjusted for age, gender, BMI, and socio-economic status.*

*Figure S3. Variations regarding the relationship between nature indices, buffer types, and buffer distances across age for standing long jump distance.*


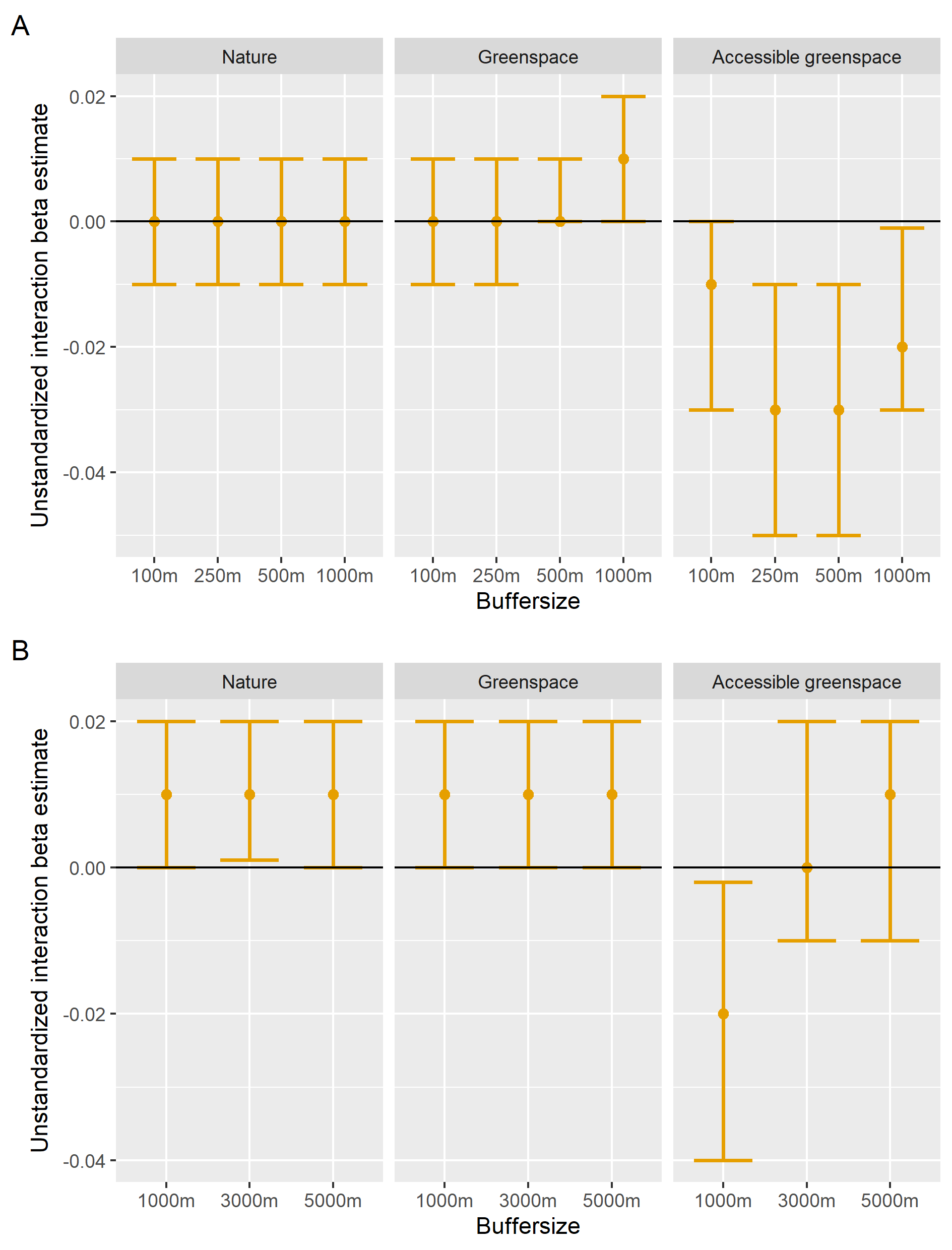


*Panel A: Unstandardized interaction beta estimates for circular buffers. Panel B: Unstandardized interaction beta estimates for street-network buffers. Sample size: N = 2,493. Error bars represent 95% confidence intervals. All models were adjusted for age, gender, BMI, and socio-economic status.*

*Figure S4. Variation regarding the relationship between nature indices, buffer types, and buffer distances for mental health problems across youth with low and high socio-economic status compared to youth with medium socio-economic status.*


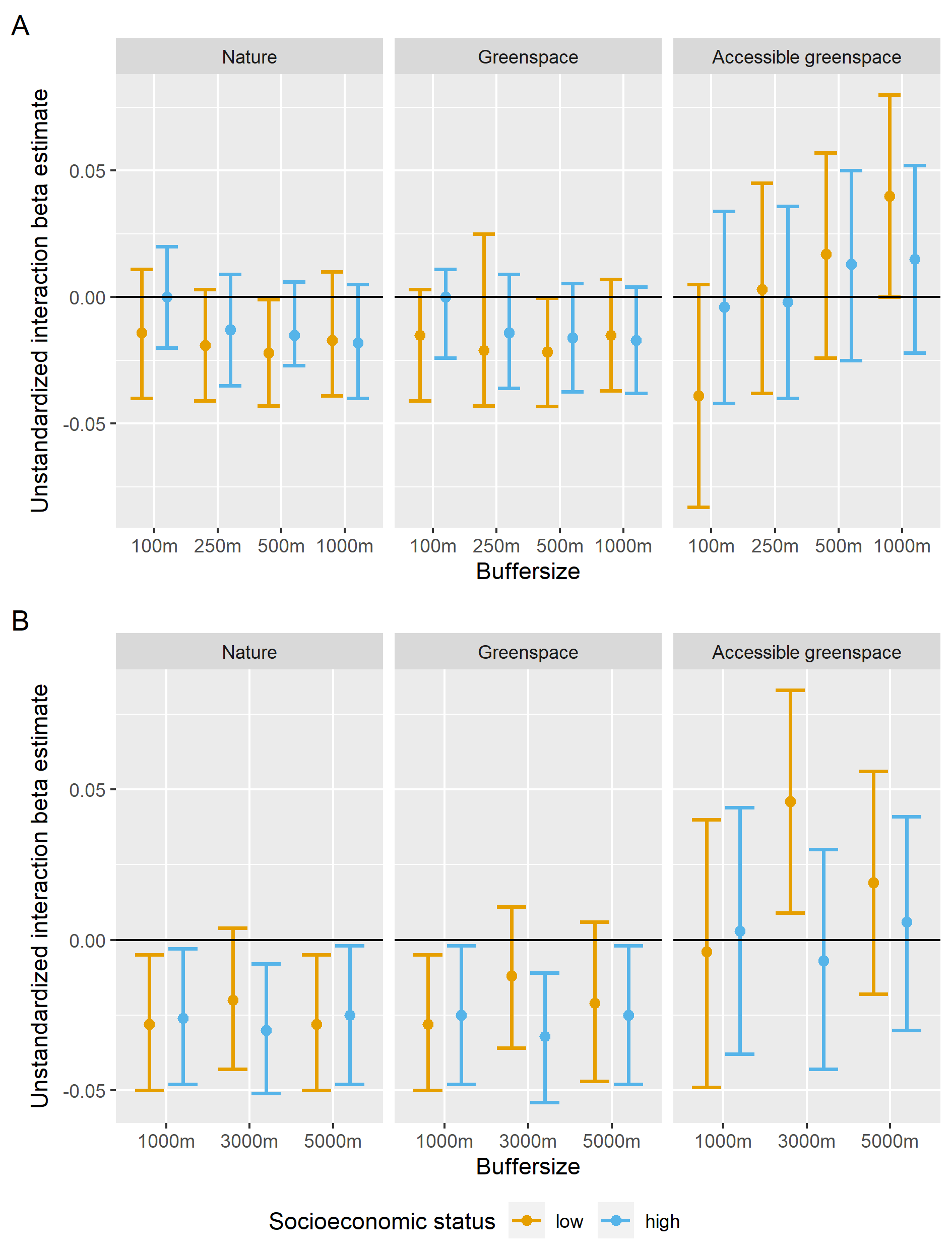


*Panel A: Unstandardized interaction beta estimates for circular buffers. Panel B: Unstandardized interaction beta estimates for street-network buffers. Sample size: N = 2,341. Error bars represent 95% confidence intervals. All models were adjusted for age, gender, BMI, and socio-economic status.*

*Figure S5. Variation regarding the relationship between nature indices, buffer types, and buffer distances across age regarding mental health problems.*


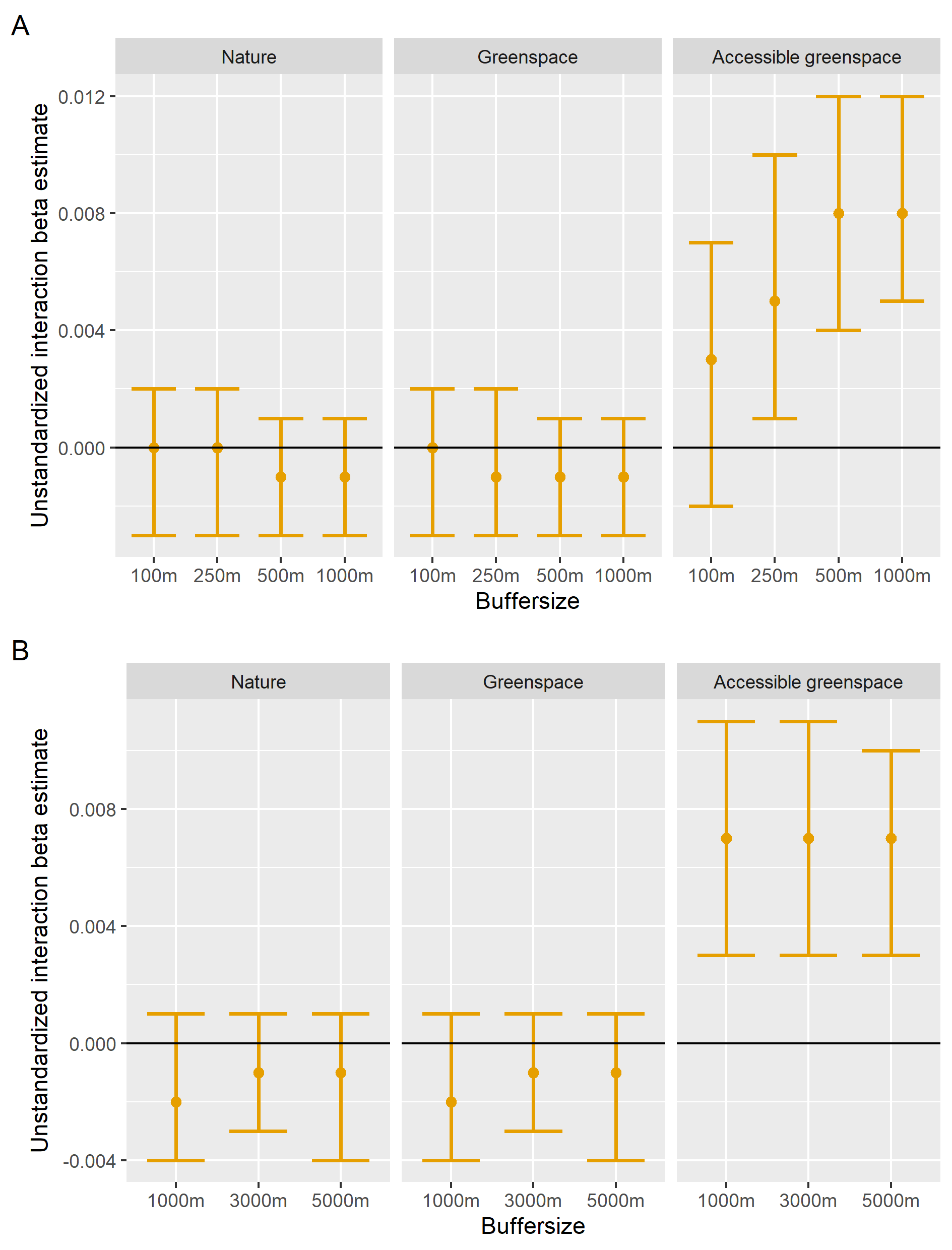


*Panel A: Unstandardized interaction beta estimates for circular buffers. Panel B: Unstandardized interaction beta estimates for street-network buffers. Sample size: N = 2,341. Error bars represent 95% confidence intervals. All models were adjusted for age, gender, BMI, and socio-economic status.*
